# Secure top most significant genome variants search: iDASH 2017 competition

**DOI:** 10.1186/s12920-018-0399-x

**Published:** 2018-10-11

**Authors:** Sergiu Carpov, Thibaud Tortech

**Affiliations:** grid.457331.7 CEA, LIST, Point Courrier 172, Gif-sur-Yvette Cedex, 91191 France

**Keywords:** Genome variants search, Genomic data privacy, IDASH competition, Intel SGX

## Abstract

**Background:**

One of the 3 tracks of iDASH Privacy & Security Workshop 2017 competition was to execute a whole genome variants search on private genomic data. Particularly, the search application was to find the top most significant SNPs (Single-Nucleotide Polymorphisms) in a database of genome records labeled with *control* or *case*. In this paper we discuss the solution submitted by our team to this competition.

**Methods:**

Privacy and confidentiality of genome data had to be ensured using Intel SGX enclaves. The typical use-case of this application is the multi-party computation (each party possessing one or several genome records) of the SNPs which statistically differentiate control and case genome datasets.

**Results:**

Our solution consists of two applications: (i) compress and encrypt genome files and (ii) perform genome processing (top most important SNPs search). We have opted for a horizontal treatment of genome records and heavily used parallel processing. Rust programming language was employed to develop both applications.

**Conclusions:**

Execution performance of the processing applications scales well and very good performance metrics are obtained. Contest organizers selected it as the best submission amongst other received competition entries and our team was awarded the first prize on this track.

## Background

In this paper we describe the solution submitted by our team to the second task of iDASH Privacy & Security Workshop 2017 competition [1]. Before proceeding to solution description itself we start by introducing some background and related works. Afterwards we describe more formally competition problem together with a typical use-case.

### Related works

DNA is the molecule that stores genetic instructions used by any living organism in their growth, development and functioning. The DNA molecules are organized in chains which form the genome. Studying human genome has plenty of practical applications in the medical, social, legal fields, etc. Any two individuals share about 99.9*%* of their genomic DNA and the remaining 0.1*%* track the differences between them. The vast majority of these differences take the form of single-nucleotide polymorphism (SNP). A SNP is a substitution of one base pair at a certain location when compared to a reference genome. Genome SNP variations are studied in order to track disease genes or heritable traits.

One important genomic application is the search for top most significant SNPs, in a dataset labeled with control and case, which are chosen according to the statistical *χ*^2^ test.

As an example, this application can be used to detect genome differences between a group of persons which has a disease and another group which does not have it. The most significant SNPs (supposedly) influence disease susceptibility.

Genome sequencing cost decreases each year [2]. More and more genome data is available for full scale medical research [3]. Cloud storage and computing is a straightforward solution to the challenge of storing and processing huge amounts of genomic data [4]. However, outsourcing genomic data to an untrusted cloud environment can be difficult or even impossible because of privacy and confidentiality concerns [5, 6]. Many research works [7–9] study the inference of sensitive personal information (e.g. person identity and appearance, disease condition) from genomic data.

Homomorphic encryption is a solution which can ensure genomic data privacy while being able to perform computations. Homomorphic property of group based cryptography was stated in [10]. The first fully homomorphic encryption scheme (supporting both addition and multiplication) was introduced by Gentry in [11]. Since then, several other authors proposed new and more efficient homomorphic encryption schemes [12–14]. The most recent one [15] being able to execute a 2-input Boolean gate in less than 13 milliseconds. On a side note, this encryption scheme was used by 2 teams in the third track of iDASH 2017 competition [1]. From an applicative point of view, the authors of [16–20] introduced and discussed the use of homomorphic encryption to genomic data processing (e.g. genetic association, logistic regression, genomic medicine). Secure multi-party computation protocols can also be used to provide private genomic data analysis [21–23]. The main issue of these solutions is the performance bottleneck when applied to large-scale genomic data computations.

Hardware assisted privacy preserving solutions (i.e. Intel Software Guard Extensions (SGX)) allow to leverage the performance gap of cryptography only based solutions (e.g. homomorphic and functional encryption, multi-party computation protocols, etc.). Intel SGX allows to pragmatically instantiate diverse cryptographic concepts without huge overhead. Secure genomic computations using Intel SGX have been studied in many research works: rare disease analysis [24], genomic queries [25], etc. The 2017 iDASH competition second track was to perform a whole genome variants search in a multi-party context.

### Overview of intel SGX

**Fig. 1:**
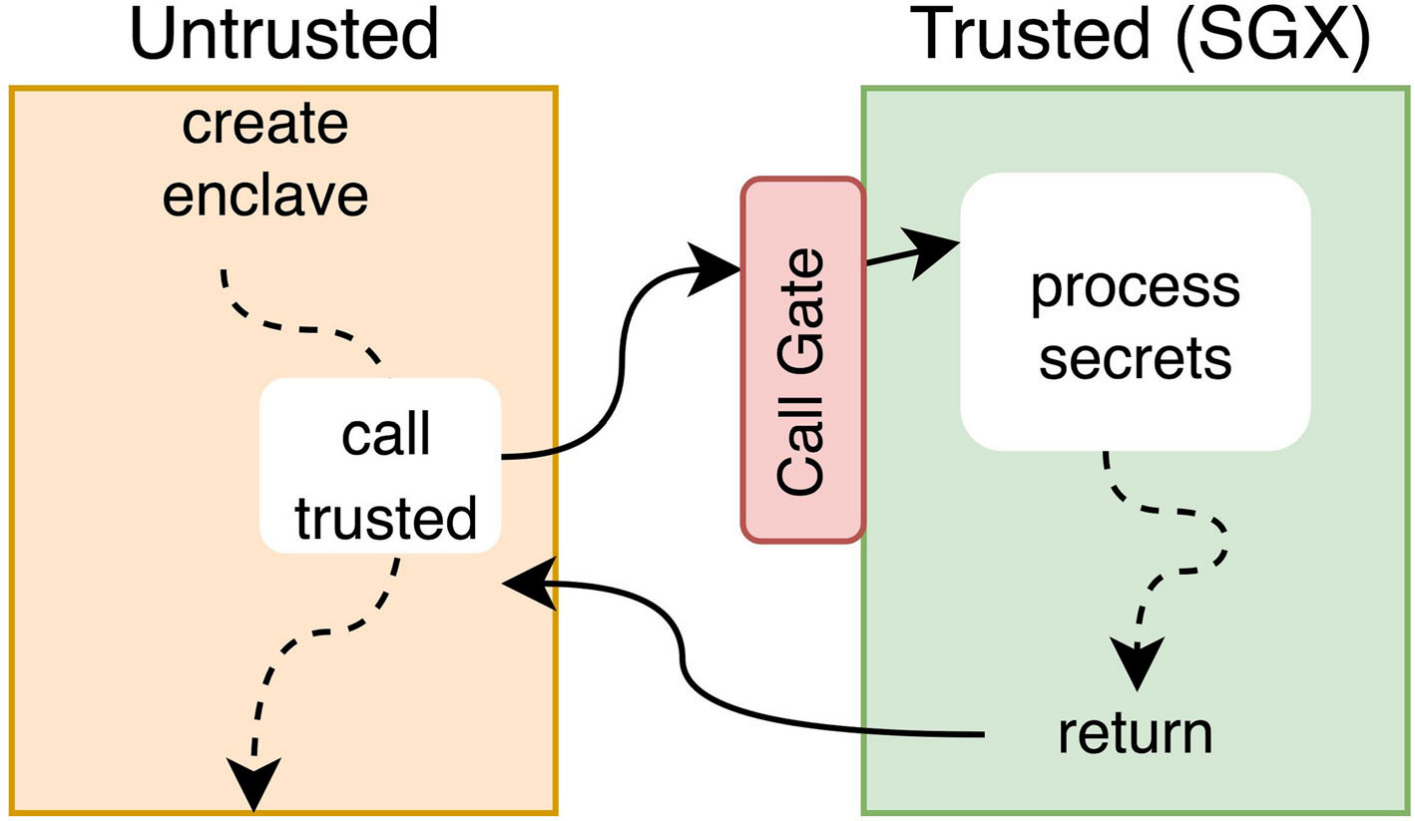
Overview of an SGX application runtime
- a Memory Encryption Engine (MEE) to encrypt/decrypt on the fly,
- a MEE buffer of 128MB, in which 96MB are available to the application.

Intel’s Software Guard Extensions (SGX) was first introduced in 2015 on the Skylake micro-architecture. The aim of this extension is to provide a Trusted Execution Environment (TEE) in which applications can protect critical code and data against malicious privileged system code (operating system, hyper-visor, BIOS, etc.). The trusted part of the application is called an enclave in SGX dialect. The key point is that enclave code and data inside the CPU perimeter runs in the clear, but are encrypted outside. Figure 1 illustrates the execution of an application using SGX. SGX is built on three components:

- 17 new CPU instructions,

More information on Intel SGX can be found in the white-paper [26] and a detailed description [27]. Possible use-cases of SGX applications are secure remote computation, secure web browsing, digital rights management, etc.

Even if at first view one can think that Intel SGX allows to securely execute applications on encrypted data, particular attention should be paid to the manner applications are implemented. Existing works [28–30] present side-channel (cache timing, page faults, memory access patterns) attacks on SGX enclaves. They arrive to discover secrets (e.g. secret key of an encryption algorithm) from applications executed inside an enclave. This attack is possible because of the information which leaks from application execution and highly depends on how the application was implemented.

### VCF file format

The Variant Call Format (VCF) is a format of text files used for storing genome variations. Compared to other file formats which store lots of redundant data (as mentioned earlier 99.9*%* of genome is shared between individuals), a VCF file tracks only differences from a reference genome. In this work we suppose that VCF files contain only SNP gene differences. A sample of VCF file (first 8 lines) is given below:

##real id in 1000genome project: HG00253

#CHROM POS ID REF ALT QUAL FILTER TYPE

1 13110 rs540538026 G A 100 PASS heterozygous

1 13116 rs62635286 T G 100 PASS heterozygous

1 13118 rs200579949 A G 100 PASS heterozygous

1 14930 rs75454623 A G 100 PASS heterozygous

1 15211 rs78601809 T G 100 PASS homozygous

1 18849 rs533090414 C G 100 PASS homozygous

A VCF file contains meta-information lines (starting with two “#” symbols), one header line (starting with a “#” symbol) and then one data line per SNP. Each SNP information line contains exactly 8 fields. First 5 fields are: chromosome identifier (CHROM), position within chromosome (POS), unique SNP identifier (ID), reference (REF) and alternate (ALT) base. We consider that chromosome and position fields are integers. SNP identifier is a string. Reference and alternate base are non equal symbols from the set {A,C,G,T,N}. The last field (TYPE) shows whether SNP is heterozygous or homozygous. One important property of VCF files is that SNPs are sorted in increasing order by chromosome and position.

## Methods

### Use-case

An important step towards better understanding of human genome is the share of genomic data between entities possessing genome databases (research institutions, state agencies, etc.). This does not necessarily imply an actual share of genomic databases between two or more entities, which can be a cumbersome and even impossible due to legal restrictions. Legislation in many countries impose a strict regulation on human genome privacy and confidentiality when storing, sharing and manipulating genomic databases. It can materialize itself in carrying out analyzes on a joined view of individual databases and sharing only the results of these analyses. The whole genome variants search for the top most important SNPs introduced previously is a good example of such analysis. Obtained results will have smaller statistical error because of a larger size input dataset when compared to an analysis performed over individual datasets.

**Fig. 2:**
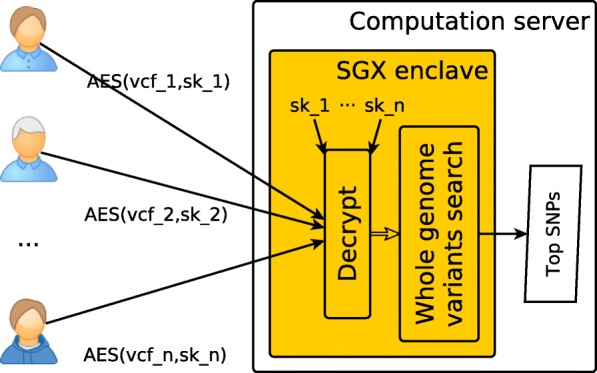
Use-case for multi-party search over encrypted genomic data

Figure 2 illustrates a typical use-case of computing the top most important SNPs for several entities. *n* actors possessing genomic data files (VCF format for illustration) are involved in this use-case. Confidentiality of genomic data is ensured by dedicated hardware (Intel SGX – introduced in the previous sub-section). The process starts with establishing trust in the computation server, namely the SGX enclave. The SGX enclave proves its authenticity to an actor, a key-exchange protocol (e.g. Diffie–Hellman) is used to establish a shared secret (a symmetric encryption key *sk*_*i*_, *i*∈1…*n*) between the actor and the enclave. Once a trusted communication channel is established each actor sends its encrypted VCF files to the computation server. In our case, AES encryption with 128-bit keys in GCM (Galois/Counter Mode) mode is used. The GCM mode provides both data authenticity (integrity) and confidentiality.

### Application: top most important SNPs search

The algorithm (a high-level view) for finding the top *K* most important SNPs in a genomic dataset labeled with control and case consists in the following steps:

1. compute SNP presence counters CTRL_CNT and CASE_CNT in control and respectively case VCF files,
2. compute the *χ*^2^ statistic for each found SNP,
3. return the top *K* most important SNPs.

CTRL_CNT and CASE_CNT are maps which associate to each SNP an integer value designating the number of control and respectively case VCF files this SNP is found in. For example CTRL_CNT[SNP] gives the number of control VCF files containing genome difference SNP. We recall that homozygous SNPs count twice in these presence maps.

#### *χ*^2^ statistic

The Pearson’s *χ*^2^ test allows to determine if there is significant difference between observed and expected frequencies in one or more categories. In the context of genome search use-case the *χ*^2^ test is used to find which SNP distributions have the largest evidence of statistical difference between case and control datasets.

Let *s* be a SNP found in a VCF file. Let *n*_*ctrl*_ and *n*_*case*_ denote the number of times SNP variation *s* appears in control and respectively case files (zero if not present). We have *n*_*ctrl*_=CTRL_CNT[*s*] and *n*_*case*_=CASE_CNT[*s*]. Let *N*_*ctrl*_ and *N*_*case*_ be respectively the number of control and the number of case files multiplied by 2 (as homozygous counts twice). SNP *s* observed frequencies *O* and expected frequencies *E* are given by:

$$\begin{array}{*{20}l} O & =\left[n_{ctrl},n_{case},N_{ctrl}-n_{case},N_{case}-n_{case}\right]\\ E & =\left[N_{ctrl}\cdot f,N_{case}\cdot f,N_{ctrl}\cdot\left(1-f\right),N_{case}\cdot\left(1-f\right)\right] \end{array} $$

Here, $f=\frac {\left (n_{ctrl}+n_{case}\right)}{\left (N_{ctrl}+N_{case}\right)}$ is *s* frequency in both datasets.

The *χ*^2^ test statistic value is equal to $\sum _{i}\frac {\left (O_{i}-E_{i}\right)^{2}}{E_{i}}$. The *p*-value is the probability that a random variable following a *χ*^2^ distribution will be larger than the above test statistic value (i.e. the survival function). The SNPs which have the largest *p*-values are those whose distributions differ the most in case and control datasets. To find the top *K* most significant SNPs one needs to compute *p*-values for each SNP in input genome dataset and to return *K* SNPs with largest *p*-values.

## Results

### Software architecture

In this sub-section we describe the global software architecture. We limit the discourse to genome processing part only and ignore the key-exchange part. Thus, in what follows we suppose that actors trust the SGX enclave and that the enclave has all the decryption keys.

We split the genome processing use-case operation in two parts: (i) compress and encrypt input genome data files and (ii) build CTRL_CNT and CASE_CNT maps and compute top *K* most important SNPs (denoted the *processing* part). Each part is implemented in a separate application. The first application (compress and encrypt) is performed by the actors possessing VCF files and the second one by the enclave. In the following subsections we discuss in more details these applications.

#### Compress & encrypt

As expected, this application compresses and encrypts a VCF file given as input. The compression step consists in rewriting SNPs from a text format into an equivalent binary format. Each VCF file SNP (i.e. a data line) is packed into 80 bits (10 bytes). Table 1 gives more information about the number of bits allocated to each SNP data line field. This compression is adapted to the specific VCF file format used in the contest and becomes lossy when the generic VCF format is used.

**Table 1:** Binary SNP format (in bits)

CHROM	POS	ID	REF & ALT	TYPE
5	32	37	5	1

Total size is 10 bytes

Before proceeding to encryption a given number of contiguous SNPs are grouped into blocks. Block binary SNPs are encrypted using AES in GCM mode. The format of a block of encrypted SNPs is given in Table 2. Here, the first field gives the number of SNPs in the block. IV is a random nonce used so that same input block of SNPs generates a different ciphertext. MAC is the message authentication code output of AES-GCM encryption needed to prove block authenticity. Followed by the encrypted stream of binary formatted SNPs.

**Table 2:** SNP block format (in bytes)

SNP count (*n*)	IV	MAC	SNP 1	...	SNP *n*
4	12	16	10	...	10

Total size is 32+10·*n* bytes

In a compressed VCF file all the blocks contain the same number of SNPs, except for the last one. Compressed and encrypted files are smaller when compared to initial ones, therefore the network traffic between the actors and the computation server is also lower. Another advantage is that using binary format input files, inside an enclave, is less prone to side-channel information leakage.

#### Processing

Searching for the top most important SNPs starts once all of the encrypted VCF files are received by the computation server. In the high-level algorithm given in previous section, during first step SNP counters CTRL_CNT and CASE_CNT are computed. All input VCF files must be read through before these maps are completely filled in and can be used to compute *χ*^2^ statistic *p*-values.

We notice that for computing the *χ*^2^ statistic for a given SNP *s* we need only the presence counters for this SNP (i.e. map values CTRL_CNT[*s*] and CASE_CNT[*s*]). The idea of horizontal partitioning the computation follows from the previous remark. So instead of filling presence maps for all the SNPs, they are only filled for a small range of SNPs. The *χ*^2^ statistics are calculated for these SNPs. A list of top most significant SNPs is updated as a function of obtained *p*-values. Afterwards this procedure is repeated for a new range of SNPs.

The main advantage of horizontally partitioning the treatment is that SNP maps (CTRL_CNT and CASE_CNT) size stay small. A drawback is that input VCF files need to be accessed several times. The fact that SNPs are ordered (by chromosome and position) in input VCF files allows to reduce the number of VCF files accesses.

**Fig. 3:**
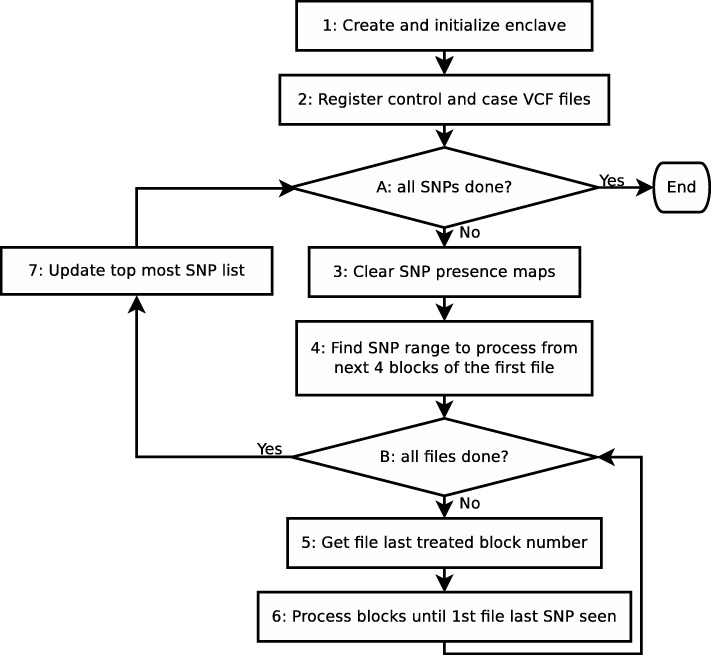
Processing algorithm block diagram

The block diagram of the processing application is shown in Fig. 3. The process starts with enclave creation and initialization (step 1). In step 2 enclave registers case and control VCF files. It simply memorizes VCF file identifiers together with boolean flags indicating whether this file belongs to case or control dataset. Horizontal partitioning of input dataset is performed in the main loop (label “A”) of the application.

During each main loop iteration a specific range of SNPs is processed. The 4 blocks of the first VCF file serve as reference range (step 4 in diagram). Thus, the SNP range to treat starts at the first SNP from the first block and ends at the last SNP of the fourth block. In the inner loop (label “B”), the SNPs belonging to the reference range from each VCF file are used to update SNP presence maps: CTRL_CNT for control files and CASE_CNT for case files. Last treated block index for each VCF file is memorized so that the next time (next main loop iteration) the application knows which block to start with.

Once all VCF files have been treated *χ*^2^ statistic *p*-value is computed for each SNP in maps CTRL_CNT and CASE_CNT. The global list of top *K* most important SNPs is adequately updated as a function of newly obtained SNP *p*-values (step 7 in diagram). Main loop is executed till all SNPs have been treated.

### Implementation language and tools

Rust programming language was used to implement these applications. Instead of the C/C++ framework provided by Intel, a Rust framework for programming SGX based applications is used.

#### Rust programming language

Rust is a new system programming language supported by Mozilla research. The aim of Rust is to be a language for highly concurrent and safe systems. Unlike C/C++, Rust has been designed with safety in mind from ground up. Mozilla describe Rust as a “safe, concurrent and practical language”. The performance of idiomatic Rust programs is comparable to ones written in C/C++. The most important strengths of Rust as a programming language are:

- zero-cost abstraction,
- type safety,
- guaranteed memory safety,
- threads without data races.

Rust is influenced by safe functional programming languages like Haskell and OCaml, and its syntax is very close to the ML family languages.

#### Rust-SGX SDK

Intel provides a Software Development Kit (SDK) for implementing SGX application. This SDK is a set of libraries and tools that allow developers to write and debug SGX applications using C/C++ language. As previously said C/C++ are unsafe languages. Developers should be very careful when implementing SGX applications in C/C++ in order to prevent memory bugs (buffer overflow, use-after-free, phantom references, etc.), which could lead to vulnerabilities and thus compromise enclave application security. Using Rust it is possible to circumvent this pitfall without sacrificing execution performance.

Rust-SGX SDK [31] is a framework that allows to implement SGX applications in Rust. This framework is developed by Baidu-X lab and is available at [32]. The framework provides a preconfigured docker image which easies its use.

## Discussion

In this section we describe in more details the applications we have implemented and the obtained execution results on a sample genomic dataset. The sample dataset has 1000 case and 1000 control VCF files. The size of dataset is approximatively 27 GB. It was provided by contest organizers for testing purposes. Final evaluation datasets were similar to this one.

All the applications have been executed on a 5-th generation Intel(R) Xeon(R) CPU E3-1240 (3.50GHz) processor with 16 GB of RAM memory and an SSD disk.

### Compress & encrypt

The first implemented application builds a compressed and encrypted version of each VCF file given as input. It starts by parsing a given number (i.e. block size) of VCF data lines, encoding them in binary format and encrypting them using AES. We recall that each data line is an SNP variation. In our study, we have chosen to encode 2080 SNPs per block, 2080 being a common multiple of binary format SNP size (10 bytes) and AES block size (16 bytes). A block of SNPs has a size of approximatively 20 KB. OpenSSL library [33] is used to perform AES encryptions.

The compress and encrypt application uses 4 threads. Each thread treats an input VCF file. Newly obtained encrypted VCF files are written to disk. Disk input/output bandwidth is the bottleneck of this application, which has to read 27 GB and write 5.5 GB. When an SSD disk is used to store output files the execution time is approximatively 65 s. We have also tested to output files to a RAM disk. In this case execution time dropped to 50 s, representing a 23% gain.

### Processing

The processing application has two binary modules, one (the main application) is executed in the public domain and other (enclave application) in the protected domain. Enclave binary module is signed, which ensures that only authenticated modules are executed by the SGX extension. As said earlier, we ignored the key-exchange phase and the multi-party computation flavor of the studied use-case. A single AES decryption key is hard-coded into the enclave application. Communication between main application and enclave is done through a light interface (*ecall* functions in SGX terms)

- encl_init loads and initializes enclave binary module,
- encl_register registers a given list of VCF files labeled with control and case,
- encl_begin starts treatment of new SNP range (main loop iteration start in Fig. 3),
- encl_run treats a SNP range for a VCF file (inner loop labeled “B”),
- encl_end ends main loop iteration.

Enclave application is executed on 8 threads. Each thread treats a SNP range from a file (i.e. inner loop in the block diagram). A custom thread-safe hash map is used for counting SNP variations. The hash map is cleared when treatment of a new SNP range begins (in ecall function encl_begin). After SNP variations from all VCF files have been added to the hash map (ecall encl_run) the output list of most important SNPs is updated (ecall encl_end). *χ*^2^ statistics are computed for each SNP variation using a leakage free numerical integration algorithm (described in following subsection). Finally, using these statistics values the global list of top K most significant SNPs is updated.

The execution time of the processing application is less than 7 sec! The main part of the execution time is due to VCF file reading. No significant differences were observed when either SSD or RAM disk was used as input medium.

Hereafter, we describe two important building blocks used in the processing application. In particular, a lock-free hash-map we have implemented to count SNP variations (CTRL_CNT and CASE_CNT) and the computation of *χ*^2^ statistic using numeric integration. Both building blocks were designed to leak as little information as possible about encrypted data (hide SNP position and contents).

#### Thread-safe hash table

SNP variations are counted and stored in associative maps CTRL_CNT and CASE_CNT. The key of these maps is built from SNP fields: chromosome number, variation position, reference and alternate base. These fields uniquely identify a SNP variation. Hash maps (in the text we use hash table term also) are used as implementations for SNP variations counters. A simple Fibonacci hashing method is used to map a SNP identifier to the hash table space. Hashes are XOR-ed with a random value, generated at enclave application start, in order to minimize information leakage from memory access patterns of sequel enclave application executions.

Rust standard library hash table implementation (std::collections::HashMap) is not thread safe. A synchronization mechanism (e.g. mutex) is needed for write accesses. One can synchronize write accesses at the global hash map level. The issue of this solution is that the whole hash map is blocked during a write operation and thus a single thread only will be able to use it. In our solution, we have implemented a hash map from scratch where synchronization is done at element level. With this implementation each thread is able to write/update the hash map in parallel.

In our implementation the hash map is an array of *N* elements indexed by the hash of SNP identifier. *N* is chosen such that the hash map size is lower than processor L3 cache size (8MB in our case). In our application hash map can store up to *N*=282914 SNPs (approximatively 6630KB). This size was empirically chosen in order to minimize hash map memory reallocations (in performed tests no reallocation is needed) and to have a reasonable fill ratio (≈50−60*%*). Each hash map element contains 3 fields:

- state of the current element,
- element key (SNP identifier),
- element value (SNP variation count).

An element can be in one of the following states (given by field state):

- empty – element is empty and available for new entry,
- update – ongoing entry element creation or value update,
- wait – entry is initialized and can be updated.

At the beginning of the execution all hash table elements are in empty state. When a new SNP entry is added or an existing SNP is updated, update state is used to synchronize concurrent threads trying to access this element. State wait means that element is free to be updated.

#### Leakage free *χ*^2^ statistic computation

The *χ*^2^ statistic *p*-value computation is performed in two steps:

- statistic value is computed from observed and expected SNP frequencies,
- *χ*^2^ distribution survival function is evaluated to obtain the *p*-value.

The survival function is a strictly decreasing function. Finding SNPs with highest *p*-values (i.e. top most important ones) is equivalent to finding SNPs which have the lowest *χ*^2^ statistic values. In our implementation the top most important SNPs list is updated (step 7 in block diagram from Fig. 3) according to SNP *χ*^2^ statistic value. The *p*-values are computed when algorithm terminates only for the resulting SNPs.

The *χ*^2^ statistic value expression can be computed directly without leaking information on input values. On the contrary, survival function does not have a closed-form expression and must be evaluated by integrating *χ*^2^ probability density function. We have implemented a numerical integration algorithm (trapezoidal rule) for this task. In order to accelerate this computation, we store an array of precomputed survival function values and perform the numerical integration for small ranges only. Our numerical integration algorithm implementation has no information leakage and the array of precomputed values is obliviously accessed.

## Conclusions

In this paper we have discussed the solution submitted to the second track of the 2017 iDASH competition. The goal of this track was to develop an application for searching the top most important SNPs in a genomic dataset. It was requested to use Intel SGX in order to ensure the privacy and the confidentiality of genomic data.

Our discourse begins with an introduction of a global view of the solution: a typical use-case and the block diagram. Afterwards we describe some implementation details and execution results on a sample dataset. Shared memory parallelism (i.e. threads) was heavily used to increase execution performance. Aggregated execution time of compression & encryption and processing applications is about 1 min. The compression & encryption step is the heaviest part, needing approximatively 50 s (mainly due to I/O bandwidth bottleneck). In the typical use-case we have discussed about, this execution time is evenly distributed over implied parties (i.e. each party encrypts its own VCF files). Data space size of our processing application was smaller than processor’s L3 cache size which contributed a lot to minimizing the number of costly page evictions.

Intel SGX enclave mechanism is not the “holy grail” for privacy preserving computations. Several works from the literature describe side-channel attacks on SGX enclave applications, all these attacks being possible because of code vulnerability. Even if it was outside of contest’s goal, we have given a particular attention to lower information leakage from application execution in SGX enclave. We have implemented our enclave application and in particular two software blocks, hash table and *χ*^2^ statistics computation, with small information leakage.

In perspective, we think that our compression step can be further optimized. That is to say, a larger compression ratio can be obtained without limiting the functionality of the performed computation. This will allow to further optimize communication size and the performance of the processing application. Also, we think that a more formal analysis of the information which our enclave application leaks is needed in order to better understand the challenge of the outsourced computation.
